# Emergency Uterine Artery Embolization Used for Managing Profuse Intra-Abdominal Bleeding and Uterine Rupture in a Patient with Advanced Cervical Cancer

**DOI:** 10.3390/diagnostics12051081

**Published:** 2022-04-26

**Authors:** Hana Habanova, Peter Mikula, Tomas Tvrdik, Eva Dedinska, Katarina Komaromy, Igor Rusnak

**Affiliations:** 1 1st Clinic of Gynecology and Obstetrics, Slovak Medical University and University Hospital Bratislava, Limbova 5, 83305 Bratislava, Slovakia; evadedinska@yahoo.com (E.D.); katarina.gerincova@yahoo.com (K.K.); irusnak@pobox.sk (I.R.); 2 Clinic of Radiology, Faculty of Medicine of Comenius University, Slovak Medical University, Limbova 5, 83305 Bratislava, Slovakia; mikula.dr@gmail.com (P.M.); sk.tomas.tvrdik@gmail.com (T.T.); 3 Central Laboratories, Faculty of Chemical and Food Technology, Slovak University of Technology in Bratislava, Radlinského 9, 81237 Bratislava, Slovakia

**Keywords:** cervical cancer, uterine rupture, uterine artery embolization, hemoperitoneum, interesting images

## Abstract

Advanced cervical cancer can lead to life-threatening vaginal bleeding. Emergency uterine artery embolization (UAE) has been successfully used in such cases to achieve hemostasis. Our case demonstrates the unusual emergency use of this procedure to cease heavy hemorrhage, which led to hematometra, uterine rupture and hemoperitoneum in a patient with a large tumor in the cervical region. Vaginal bleeding was minimal in this case. The emergency UAE controlled the bleeding, and the patient was scheduled for laparotomy soon after the procedure, where a supracervical hysterectomy with bilateral salpingo-oophorectomy and the removal of blood and blood clots was performed. Since the tumor primarily involved the parametria, a sample was taken for histopathology examination with the following result: squamocellular HPV-associated cervical carcinoma. The postoperative management of the patient consisted of combined chemotherapy and radiotherapy, with no complications related to the UAE. Four months after the procedure the patient is reasonably well. Urgent surgery was not the optimal decision because of the alteration of the pelvic anatomy by the tumor, and thus the UAE enabled us to manage this life-threatening condition quickly, allowing us to best prepare the patient for further therapeutic modalities.

**Figure 1:**
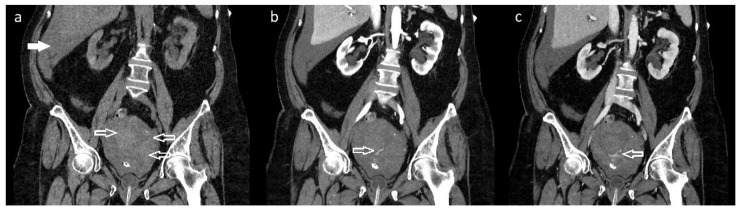
Abdominal computed tomography (CT) scan with the application of 90 mL intravenous contrast medium Visipaque 320 revealed a uterine tumor involving the cervix (size: 40 × 50 × 70 mm), hematometra with active bleeding from the tumorous mass, and a hemoperitoneum. (**a**) Non-contrast: coronal plane with hematometra in the enlarged uterus with tumor (arrows) and hemoperitoneum located in the Morison’s pouch (full arrow). (**b**) Contrast enhanced: coronal plane (arterial phase 30 s after applying the contrast agent) where an active extravasation of the contrast agent from the dorsocaudal part of the tumor can be seen (arrow). (**c**) Contrast enhanced: coronal plane (venous phase 70 s after applying the contrast agent). Continued process of the extravasation of the contrast agent, pointing to actively bleeding arterial area (arrow). A 67-year-old patient was referred to our department with a sudden onset of sharp lower abdominal pain and an episode of vaginal bleeding. The patient had a short history of intermittent, low-intensity postmenopausal bleeding and the diagnosis of pelvic mass on the ultrasound (although the diagnostic process for this was incomplete). Upon admission the patient had tachypnea, tachycardia, and diffuse abdominal rigidity. A speculum vaginal examination showed a small and extremely hard to visualize portion of ectocervix and, by bimanual palpation, a tumorous mass (involving both cervix and parametria) was diagnosed (minimal vaginal bleeding present). An urgent computed tomography scan was ordered which revealed an enlarged uterus with a tumor in the cervical region and active extravasation in the arterial and venous phase after applying the contrast, which pointed to active arterial bleeding from the lesion causing hematometra and subsequently uterine rupture and hemoperitoneum. We considered an acute surgical intervention as an unfavorable option because it was a locally advanced disease which had altered the pelvic anatomy in an already hemodynamically unstable patient. There was also a significant drop in hemoglobin levels (123 g/L vs. 100 g/L samples taken in approximately 1.5 h interval), which pointed to a major blood loss. We were informed about the possibility of a UAE and, after the required initial management and blood transfusions, the patient was transported to the angiography department.

**Figure 2:**
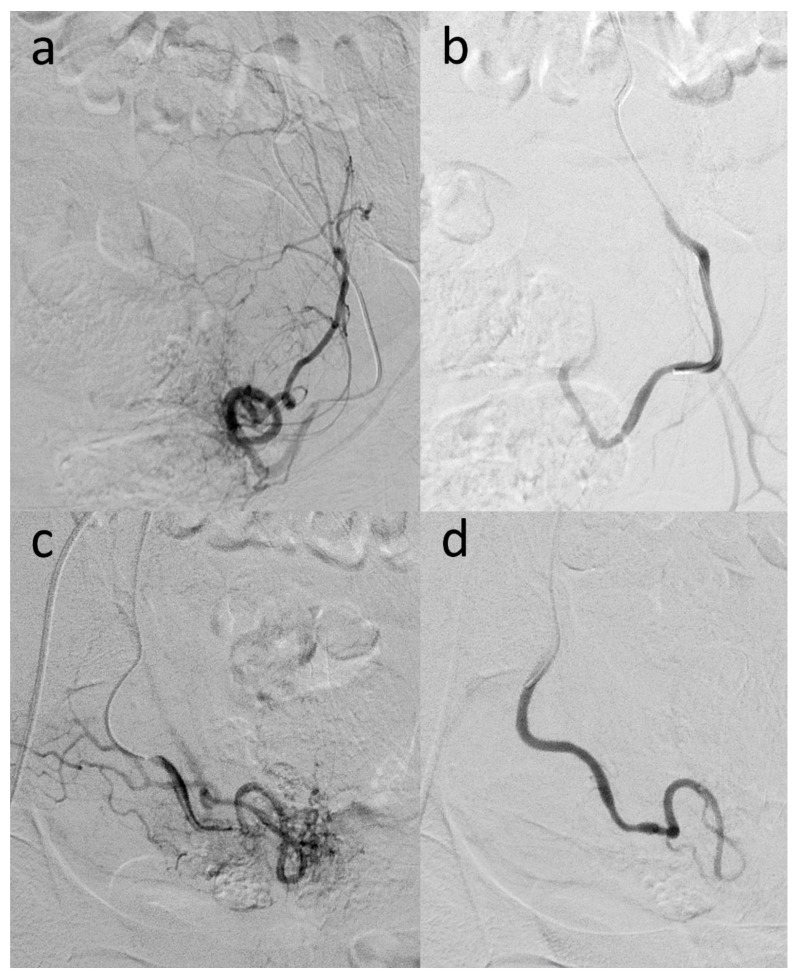
The UAE procedure was initiated with an ultrasonography-controlled transfemoral retrograde approach under local anesthesia. The left internal artery was catheterized with the cross-over technique. After selective angiography, the left uterine artery was visualized (**a**), catheterized, and selectively embolized with Embozene Microspheres™ (400 μm particle size) (Boston Scientific, Marlborough, MA, USA) until the stagnation of blood flow occurred (**b**). Subsequently, the right internal iliac artery and, super selectively, the right uterine artery were catheterized. After selective visualization of the hyper vascularized uterine zone (**c**), it was embolized with Embozene Microspheres (250–400 μm particle size) (Boston Scientific, Marlborough, MA, USA) until the stasis of blood flow was attained (**d**). Angiographic control was performed, and no flow was seen in either of the uterine arteries. No other vascularization was affected. The patient was returned immediately after the procedure to the gynecologic department.

**Figure 3:**
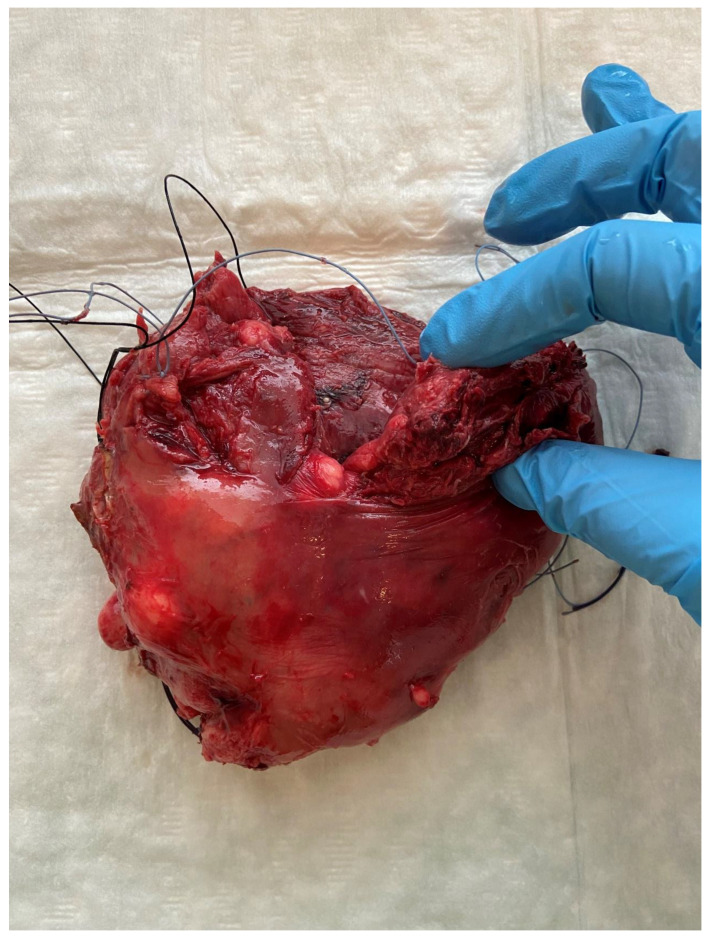
The next day, a laparotomy revealed 2100 mL of dark blood and blood clots in the abdominal cavity, and a complete uterine rupture in the fundus was diagnosed (Figure 3). The tumor of cervical origin largely involved the parametria; therefore, a supracervical hysterectomy with bilateral salpingo-oophorectomy was performed and an excision from the mass (17 × 12 × 15 mm) was sent for histopathological examination which confirmed the diagnosis of invasive squamocellular HPV-associated cervical carcinoma. Subsequent management of the patient consisted of concomitant chemotherapy (cisplatin regimen) and radiotherapy, and no significant vaginal bleeding was experienced after the embolization procedure. UAE is an effective tool to eliminate uterine bleeding in various gynecologic and obstetric conditions [1]. There are scattered reports of UAE use in cases of inoperable cervical cancer, in attempts to control massive vaginal hemorrhages where a conservative approach (the administration of hemostatic agents, vaginal packing, and blood transfusions) is not adequate to achieve hemostasis. For these patients, a great loss of blood is often a life-threatening condition [2,3]. It is important to emphasize the use of UAE, if possible, because the procedure provides detailed visualization of the vessel causing the hemorrhage and allows minimally invasive, direct therapy to achieve hemostasis. There are complications associated with UAE; however, the majority of these are of minor grade (in most cases postembolization syndrome is treated symptomatically). Severe complications of UAE can be lessened by comprehension of the vascular anatomy and the super selective embolization of the uterine arteries [3].
